# Higher divorce risk when mates are plentiful? Evidence from Denmark

**DOI:** 10.1098/rsbl.2018.0475

**Published:** 2018-09-26

**Authors:** Caroline Uggla, Gunnar Andersson

**Affiliations:** Stockholm University Demography Unit (SUDA), Sociology Department, Stockholm University, 106 91 Stockholm, Sweden

**Keywords:** adult sex ratio, divorce, sex differences, occupational sector, Denmark

## Abstract

Work from social and biological sciences has shown that adult sex ratios are associated with relationship behaviours. When partners are abundant, opportunities for mate switching may increase and relationship stability decrease. To date, most of the human literature has used regional areas at various levels of aggregation to define partner markets. But, in developed countries, many individuals of reproductive age spend a considerable amount of time outside their residential areas, and other measures may better capture the opportunities to meet a (new) partner. Here, we use Danish register data to test whether the sex ratio of the occupational sector is linked to divorce. Our data cover individuals in Denmark who married during 1981–2002 and we control for age at and duration of marriage, education and parity. Results support the prediction that a higher proportion of opposite-sex individuals in one's occupational sector is associated with higher divorce risk. This holds for both men and women, but associations are somewhat stronger for men and vary by education. Our results highlight the need to study demographic behaviours of men and women simultaneously, and to consider partner markets beyond geographical areas so that differing strategies for males and females may be examined.

## 1. Introduction

The ratio of males to females in a population has been linked to various mating and parenting behaviours. Relationship stability is one such factor that has been explored in studies of animals and humans alike. Divorce and infidelity in shore birds are more common in female-biased than male-biased species [1] and across human societies, and evidence suggests that when women are scarce, long-term pairbonds are more likely to be formed and relationships are more stable [2–4]. This implies that it is when *men* have more options to find another partner that relationships are more likely to dissolve. Less work has considered whether similar patterns can be seen for females when they have many alternative mates.

Part of this shortage is due to the fact that most human studies exploring sex ratio skews have defined the mating market based on area units, from states to neighbourhoods and villages [5–7]. By definition, adult sex ratios of areas are the same for a cohabiting couple, thus hindering any comparisons of whether men and women react differently to mate scarcity or surplus. Moreover, geographical areas may be insufficient to capture an individual's actual chances of finding a partner as these chances are often highly affected by social networks. This may explain why individuals' perceptions of sex ratios in residential areas have been found to be not very accurate [8].

This study uses Danish register data to explore divorce risk as a function of the sex ratio in men's and women's occupational sector. In sociology and demography, characteristics of an individual's workplace have been used to measure mating markets [9,10]. The sector sex ratio provides a measure of the opportunities to meet opposite-sex partners in the everyday work environment. Individuals are more likely to remain within the same sector, than in the same workplace over time, especially in sectors where job switching is frequent.

Denmark is an ideal setting for this study. Divorce is broadly accepted, whether or not a couple has children, and both men and women typically stay active in the labour market after starting a family. Notably, the sector sex ratio in Denmark varies greatly: within the healthcare sector, 18% are men, whereas in construction, about 92% are men (see electronic supplementary material, table S1). Previous research from Denmark has shown that the occupational sector is linked to differences in the timing of births [11]. However, family-demographic research often focuses on women and less often studies men's reproductive behaviours. Conversely, in the biological literature, much focus has been on how *male* strategies vary with the sex ratio. The detailed Danish register data allow us to shed light on the divorce risks of all married men and women when exposed to mate scarcity or surplus at work. We predict that with more opposite-sex colleagues, both sexes will be more likely to divorce. Benefits of mate switching may be higher for males than females, but the view of stereotyped sex roles is increasingly outdated [12] and we take an exploratory approach to the magnitude of potential sex differences.

## 2. Material and methods

We use population registers comprising the entire population residing in Denmark. The data are drawn from administrative registers linked by Statistics Denmark. All information is longitudinal and available at the individual level, including various socioeconomic, demographic and civil status histories of those born in 1945 or later and employment histories from 1981 onwards. This study covers all men and women in Denmark born in or after 1945, who married an opposite-sex spouse between 1981 and 2002 and who were active in the labour market in any of those years. This renders 4 720 033 years of risk for men (102 453 divorces) and 4 954 810 years of risk for women (113 252 divorces) in employment and marriage.

Divorce risks are calculated by occupational sector (operationalized as dummies) controlling for age at marriage, duration of marriage, metropolitan residence, educational attainment and parity (table 1). Age at marriage is fixed and the other variables are time-varying. The 727 Danish sector codes are categorized into 47 occupational sectors, e.g. advertising, construction, hotel and restaurant, IT, healthcare, higher education, retail and public administration (see electronic supplementary material, table S1). Event-history analysis for time to divorce is applied (individuals are censored at emigration or death). Results are presented as relative risk ratios plotted over the proportion of men of all individuals of reproductive age (20–44 years) in the sector during 1981–2002. Because we use the entire Danish population confidence intervals are not shown.

**Table 1.** Divorce risk (1981–2002) among individuals born 1945 or later who married between 1981 and 2002. Models control for sector of occupation (see electronic supplementary material, table S1).

		time at risk (%)		relative risks (RR)	
		men	women	men	women
age at marriage (years)	16–22	5.0	14.2	1	1
	23–29	46.9	51.5	0.74	0.70
	30–39	39.4	28.1	0.67	0.67
	40 or >	8.7	6.2	0.61	0.57
residence	Copenhagen area	19.4	19.9	1	1
	rest of Denmark	80.6	80.1	0.75	0.67
highest level of education	primary	23.3	28.4	1	1
	secondary	52.2	43.9	0.71	0.66
	tertiary	24.2	27.8	0.50	0.52
parity	0	17.6	16.5	1	1
	1	26.9	25.8	0.74	0.74
	2	40.0	42.4	0.57	0.58
	3 or >	15.5	15.3	0.64	0.67
duration of marriage (years)	1	11.6	11.5	1	1
	2–3	20.5	20.4	3.73	3.53
	4–5	16.9	16.9	5.46	5.17
	6–7	13.7	13.7	5.37	4.92
	8–10	15.5	15.6	4.78	4.37
	11 or >	21.7	21.9	3.73	3.33

## 3. Results

### (a) Demographic variables

The results of the individual independent variables are in line with demographic evidence from similar countries [13]. Divorce risk is approximately 40% lower for those who married after age 40 compared to those who married aged 16–22, *ceteris paribus*. Individuals who live outside the Copenhagen metropolitan region have about 30% lower divorce risk. The highly educated have about half the divorce risk of those with lower education. The divorce risk is 40% lower for two-child parents than those with no children (table 1).

### (b) Occupational sector

**Figure 1.**
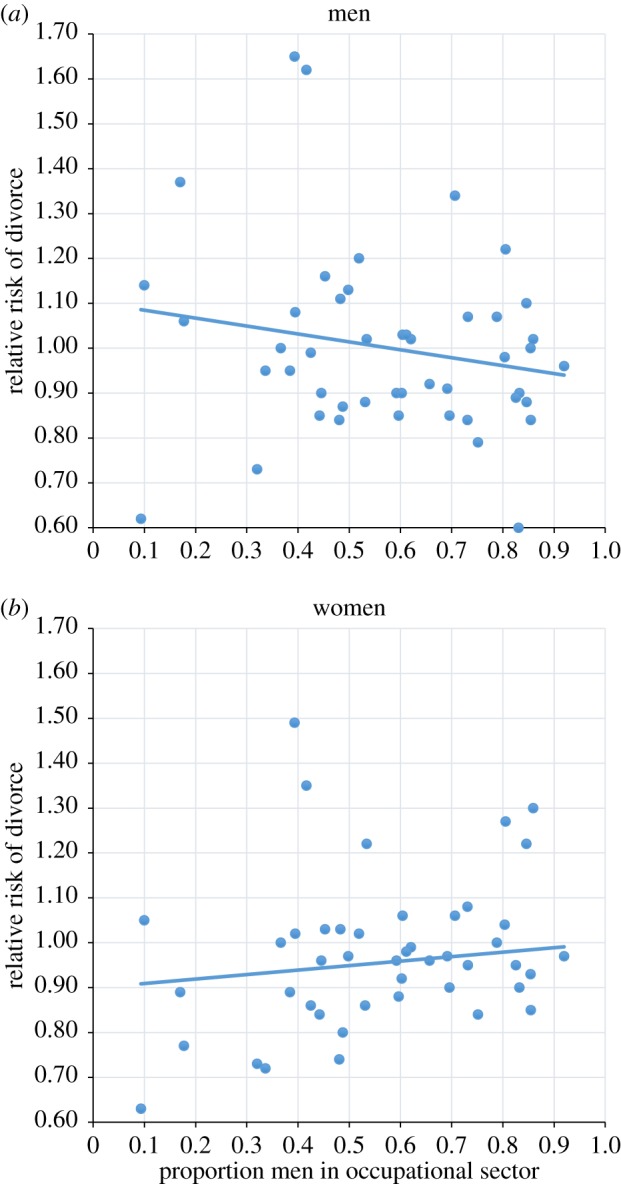
Relative divorce risks for men (*a*) and women (*b*) by the proportion of men in the occupational sector. The model includes age at marriage, duration of marriage, metropolitan residence, educational attainment and parity. (Online version in colour.)

Figure 1*a*,*b* plots the divorce risk as a function of the sector sex ratio, net of demographic controls. Results indicate that there is a negative association between the share of men in the sector and divorce risk for men, and a somewhat positive association for women. The correlation coefficients indicate that sex composition of the sector is more strongly associated with divorce risk among men than among women (*r* = −0.187 for men, *r* = 0.116 for women). The sectors associated with the highest divorce risks for both men and women are the hotel and restaurant and manpower sectors, while low divorce risks are found among men and women in farming, pharmaceutical and library sectors (see electronic supplementary material, table S1). Interactions between an individual's education and sector show variation in the associations: divorce risk–sex ratio correlations by education are *r* = −0.11 (primary), *r* = −0.23 (secondary) and *r* = −0.21 (tertiary) for men, and *r* = 0.14 (primary), *r* = 0.01 (secondary) and *r* = 0.04 (tertiary) for women. This suggests that the association is about twice the size among highly educated men compared to low educated. For women, the relationship is reversed and highly educated women have barely any increase in divorce risk in more male-biased sectors.

## 4. Discussion

We add to previous evidence that the adult sex ratio is associated with relationship dynamics by presenting evidence that more members of the opposite sex in one's occupational sector are associated with higher divorce risk in Denmark. We address the issue that area sex ratios may not accurately capture interactions with the opposite sex, and are often unable to examine whether opportunities to meet new partners differ for men and women. Results indicate that an abundance of partners of the opposite sex in one's occupational sector is more strongly associated with divorce for men, especially those with high education, while for highly educated women, the association is weak or non-existing.

Many people tend to partner with someone of the same educational level and field as themselves. Educational homogamy may be the result both of a preference for a partner with the same type of education and a higher likelihood to meet a partner similar to oneself in educational institutions and workplaces [14]. A Danish study on sex ratios of workplaces found that dissolution—but not relationship formation—was higher when partners were abundant [9] and may reflect higher search costs and narrower partner markets for those already married. A Swedish study showed that divorce risks were 70% higher in workplaces with only opposite-sex colleagues of suitable age, compared to workplaces with all same-sex individuals [10].

Some previous studies have found sex differences in behaviours linked to sex ratios in the workplace or sector, but none to our knowledge has simultaneously explored heterogeneity by education. A US study found that more opposite-sex partners in the occupational industry were associated with higher infidelity risk among men, but not among women. However, sample sizes were small and the data cross-sectional [15]. Another US study incorporated the sex ratio of the spouse's workplace and found that the sex ratio of women's workplace had a greater effect on divorce than that of her husband's, but for men, there was no effect of spouse's workplace sex ratio [16].

There are several potential mechanisms for higher mate switching when mates are plentiful. Divorce may be higher when an individual is surrounded by more individuals of the opposite sex due to higher opportunities of meeting a new partner, or because the abundance of partners influences the overall perception of alternative partner choices. The relative role of these pathways is difficult to ascertain because data on the behaviours leading up to the event, or who initiated the divorce, are often lacking. It is possible that an abundance of alternative partners for one spouse leads the other spouse to alter his/her strategy and that the combination of these factors leads to divorce.

Some caution regarding selection into a given sector is warranted. It is possible that individuals who work in an occupational sector where they are outnumbered by the opposite sex differ compared to same-sex peers and that this has bearing on divorce risk, for example, if such individuals have personality traits that increase the risk of divorce or being the minority sex is a stressor. Another possibility is that men in female-biased sectors might be more likely to divorce due to lower social status that comes with lower pay in such sectors. Moreover, it is possible that social norms may covary with the sex ratio and that this has bearing on divorce risk. While we do control for education at the individual level (a known predictor of divorce [13]), we cannot completely disregard this explanation. Our analyses concern marriages only and it is plausible that the effects seen here would be greater had we also been able to incorporate cohabiting unions, which have higher dissolution risk and are more common among the low educated [13].

Especially high divorce risks—for both sexes—in the hotel and restaurant sector and low risks in the library and farming sectors might be due to different personality types seeking to work in such sectors, different levels of stress in the work environment or the level of interpersonal interactions. However, if the opportunity for social interaction within sectors applies equally to women and men, this cannot account for the sex differences seen. More work is needed to understand how accurately sex ratios reflect real interactions with the opposite sex, both in sectors and geographical areas.

Many studies of relationship stability and sector or workplace sex ratios come from a US context, where costs and benefits of divorce as well as selection into female labour-force participation may differ from the Nordic context. We have shown that even in the egalitarian Danish setting, there is a slight gender difference as the sector sex ratio appears more strongly associated with divorce among men than women, and is barely noticeable for highly educated women. Future research should explore both partners' alternative partner options simultaneously to uncover what circumstances lead to divorce.

## Supplementary Material

Supplementary Table 1

## Data accessibility

Data access was granted by Statistics Denmark (project nr 701908). An application needs to be made to Statistics Denmark in order to access this information, due to the sensitive nature of the data.

## References

[1] Liker A, Freckleton RP, Székely T 2014 Divorce and infidelity are associated with skewed adult sex ratios in birds. Curr. Biol. 24, 880–884. (10.1016/j.cub.2014.02.059) 24656831

[2] Schacht R, Kramer KL 2016 Patterns of family formation in response to sex ratio variation. PLoS ONE 11, 1–14. (10.1371/journal.pone.0160320) PMC4996529 27556401

[3] Uggla C, Mace R 2017 Adult sex ratio and social status predict mating and parenting strategies in Northern Ireland. Phil. Trans. R. Soc. B 372, 20160318 (10.1098/rstb.2016.0318) 28760761 PMC5540860

[4] South SJ, Trent K, Shen Y 2001 Changing partners: toward a macrostructural-opportunity theory of marital dissolution. J. Marriage Fam. 63, 743–754.

[5] Uggla C, Mace R 2016 Local ecology influences reproductive timing in Northern Ireland independently of individual wealth. Behav. Ecol. 27, 158–165. (10.1093/beheco/arv133)

[6] Schacht R, Borgerhoff Mulder M 2015 Sex ratio effects on reproductive strategies in humans. R. Soc. open sci. 2, 140402 (10.1098/rsos.140402) 26064588 PMC4448795

[7] Pollet TV, Nettle D 2008 Driving a hard bargain: sex ratio and male marriage success in a historical US population. Biol. Lett. 4, 31–33. (10.1098/rsbl.2007.0543) 18055406 PMC2412948

[8] Gilbert J, Uggla C, Mace R 2016 Knowing your neighbourhood: local ecology and personal experience predict neighbourhood perceptions in Belfast, Northern Ireland. R. Soc. open sci. 3, 160468 (10.1098/rsos.160468) 28083095 PMC5210677

[9] Svarer M 2007 Working late: do workplace sex ratios affect partnership formation and dissolution? J. Hum. Resour. 42, 582–595. (10.3368/jhr.XLII.3.582)

[10] Åberg Y 2003 Social interactions: studies of contextual effects and endogenous processes. PhD dissertation Stockholm Studies on Social Mechanisms, vol. 6, Department of Sociology.

[11] Andersson G, Neyer G 2012 Gendering occupation and fertility: a comparison between women's and men's childbearing behavior by occupational branches. In European Population Conference, Stockholm, 13–16 June 2012.

[12] Brown GR, Laland KN, Borgerhoff Mulder M 2009 Bateman's principles and human sex roles. Trends Ecol. Evol. 24, 297–304. (10.1016/j.tree.2009.02.005) 19403194 PMC3096780

[13] Jalovaara M 2013 Socioeconomic resources and the dissolution of cohabitations and marriages. Eur. J. Popul. 29, 167–193. (10.1007/s10680-012-9280-3)

[14] Nielsen HS, Svarer M 2009 Educational homogamy: how much is opportunities? J. Hum. Resour. 44, 1066–1086. (10.1353/jhr.2009.0029)

[15] Kuroki M 2013 Opposite-sex coworkers and marital infidelity. Econ. Lett. 118, 71–73. (10.1016/j.econlet.2012.09.023)

[16] McKinnish TG 2007 Sexually integrated workplaces and divorce: another form of on-the-job search. J. Hum. Resour. 42, 331–352. (10.3368/jhr.XLII.2.331)

