# Supplementary material for: Higher divorce risk when mates are plentiful? Evidence from Denmark
Source: Biol Lett. 2018 Sep 26;14(9):20180475. doi: 10.1098/rsbl.2018.0475 (PMC6170753; doi:10.1098/rsbl.2018.0475)
Supplement: Supplementary Table 1 [file rsbl20180475supp1.docx]

|  | Proportion men insector | MEN | | WOMEN | |
| --- | --- | --- | --- | --- | --- |
|  |  | RR | Exposure time | RR | Exposure time |
| Farming | 0.83 | 0.60 | 205 802 | 0.55 | 53 659 |
| Mining | 0.85 | 0.88 | 10 339 | 0.56 | 1 816 |
| Food | 0.62 | 1.02 | 161 588 | 0.99 | 110 432 |
| Textile | 0.34 | 0.95 | 24 337 | 0.72 | 47 911 |
| Wood | 0.83 | 0.90 | 38 071 | 0.90 | 10 651 |
| Paper | 0.73 | 1.07 | 24 217 | 0.95 | 9 526 |
| Graphical | 0.60 | 1.03 | 67 412 | 1.06 | 46 825 |
| Chemical | 0.61 | 1.03 | 100 055 | 0.98 | 72 934 |
| Glass | 0.80 | 0.98 | 54 846 | 1.04 | 14 385 |
| Metal production | 0.79 | 1.07 | 23 687 | 1.00 | 7 990 |
| Metal work | 0.85 | 1.00 | 115 521 | 0.93 | 28 642 |
| Apparatus | 0.66 | 0.92 | 71 239 | 0.96 | 49 794 |
| Medical & optical equipment | 0.59 | 0.90 | 30 011 | 0.96 | 26 705 |
| Vehicles | 0.83 | 0.89 | 236 136 | 0.95 | 68 424 |
| Furniture | 0.70 | 0.85 | 65 184 | 0.90 | 40 724 |
| Water & heating | 0.75 | 0.79 | 44 197 | 0.84 | 13 641 |
| Construction | 0.92 | 0.96 | 461 731 | 0.97 | 48 372 |
| Car service | 0.85 | 0.84 | 139 131 | 0.85 | 29 523 |
| Wholesale | 0.69 | 0.91 | 383 776 | 0.97 | 175 786 |
| Retail | 0.43 | 0.99 | 152 021 | 0.86 | 222 104 |
| Pharmacy | 0.09 | 0.62 | 999 | 0.63 | 11 958 |
| Hotel & restaurant | 0.39 | 1.65 | 45 767 | 1.49 | 72 959 |
| Transport | 0.81 | 1.22 | 259 709 | 1.27 | 61 940 |
| Shipping | 0.71 | 1.34 | 62 385 | 1.06 | 25 972 |
| Postal services and telecom | 0.60 | 0.90 | 86 148 | 0.92 | 67 638 |
| Finance | 0.48 | 0.84 | 275 030 | 0.74 | 305 118 |
| IT (software and hardware) | 0.73 | 0.84 | 78 048 | 1.08 | 30 067 |
| Research & Development | 0.49 | 0.87 | 15 705 | 0.80 | 15 713 |
| Technical & business services | 0.60 | 0.85 | 87 521 | 0.88 | 50 950 |
| Advertising | 0.50 | 1.13 | 16 796 | 0.97 | 15 663 |
| Manpower | 0.42 | 1.62 | 7 208 | 1.35 | 16 049 |
| Cleaning | 0.45 | 1.16 | 33 966 | 1.03 | 47 965 |
| Services (other) | 0.48 | 1.11 | 32 422 | 1.03 | 35 234 |
| Public administration (ref) | 0.37 | 1 | 130 200 | 1 | 281 689 |
| Security | 0.85 | 1.10 | 190 092 | 1.22 | 49 947 |
| School | 0.38 | 0.95 | 116 165 | 0.89 | 197 829 |
| High-school | 0.39 | 1.08 | 100 485 | 1.02 | 134 249 |
| Higher education | 0.53 | 0.88 | 36 899 | 0.86 | 35 868 |
| Health care | 0.18 | 1.06 | 103 421 | 0.77 | 489 806 |
| Social care | 0.17 | 1.37 | 109 318 | 0.89 | 894 181 |
| Sewerage, garbage, recycling | 0.86 | 1.02 | 47 137 | 1.30 | 7 521 |
| Organizations (unions, political) | 0.45 | 0.90 | 52 796 | 0.96 | 64 274 |
| Media | 0.52 | 1.20 | 34 823 | 1.02 | 30 035 |
| Library | 0.32 | 0.73 | 3 782 | 0.73 | 12 091 |
| Leisure | 0.53 | 1.02 | 13 694 | 1.22 | 10 416 |
| Personal | 0.44 | 0.85 | 5 127 | 0.84 | 7 267 |
| Beauty | 0.10 | 1.14 | 2 264 | 1.05 | 24 824 |

Supplementary Table 1. Relative risks (RR) of divorce by occupational sector, and exposure time (half-months) and proportion men in the sector (age 20-44). Covers the entire married population who were residing in Denmark any time between 1981-2002.
